# Simultaneous targeting of mitochondria and monocytes enhances neuroprotection against ischemia–reperfusion injury

**DOI:** 10.1038/s41598-020-71326-x

**Published:** 2020-09-02

**Authors:** Arihide Okahara, Jun-ichiro Koga, Tetsuya Matoba, Masaki Fujiwara, Masaki Tokutome, Gentaro Ikeda, Kaku Nakano, Masaki Tachibana, Tetsuro Ago, Takanari Kitazono, Hiroyuki Tsutsui, Kensuke Egashira

**Affiliations:** 1 Department of Cardiovascular Medicine, Graduate School of Medical Sciences, Kyushu University, 3-1-1, Maidashi, Higashi-ku, Fukuoka, 812-8582 Japan; 2 Department of Cardiovascular Research, Development, and Translational Medicine, Center for Cardiovascular Disruptive Innovation, Kyushu University, Fukuoka, Japan; 3 Department of Medicine and Clinical Science, Graduate School of Medical Sciences, Kyushu University, Fukuoka, Japan; 4 Department of Translational Medicine, Kyushu University Graduate School of Pharmaceutical Sciences, Fukuoka, Japan

**Keywords:** Drug development, Preclinical research, Translational research, Cell death and immune response, Inflammation

## Abstract

Ischemia–reperfusion injury impairs the efficacy of reperfusion therapy after ischemic stroke. Cyclophilin D (CypD)-mediated openings of mitochondrial permeability transition pore (mPTP) and subsequent monocyte-mediated inflammation are considered as major mechanisms of reperfusion injury. However, no medical therapies are currently available. Therefore, we have tested a hypothesis that simultaneous targeting of mPTP and inflammation confers substantial neuroprotection after cerebral ischemia–reperfusion. To address this point, we prepared CypD knockout mice, C–C chemokine receptor 2 (CCR2) knockout mice and CypD/CCR2 double knockout mice. These mice were subjected to 60 min transient cerebral ischemia by occluding middle cerebral arteries. Neurological deficits evaluated 3 days after reperfusion were significantly attenuated in CypD/CCR2 double knockout mice as compared to wild-type mice and other single knockout mice. Then, we have prepared polymeric nanoparticles containing cyclosporine A (CsA-NPs) and pitavastatin (Pitava-NPs), targeting mPTP opening and inflammation, respectively. Simultaneous administration of CsA-NP and Pitava-NP at the time of reperfusion also decreased infarct size and attenuated neurological deficits as compared to control nanoparticles and single administration of CsA-NPs or Pitava-NPs. These results indicate that simultaneous targeting of the mPTP opening and monocyte-mediated inflammation could be a novel strategy for better neurological outcomes in patients with ischemic stroke.

## Introduction

Innovative therapeutic strategies for protecting the brain from ischemia–reperfusion (IR) injury are necessary to decrease infarct volume and improve clinical outcomes after reperfusion therapy for the treatment of ischemic stroke^1^.Within several minutes after reperfusion, opening of mitochondrial permeability transition pore (mPTP) triggers cell deaths in tissues exposed to IR injury^2^. Inflammatory monocytes, Ly6C^high^CCR2^+^ in mice or CD14^high^CD16^−^ in humans, accumulate to the cerebral tissue as early as several hours after reperfusion and also trigger cell death^3–8^.

Cyclosporine A (CsA) is a widely used drug after organ transplantation which binds to cyclophilin A and inhibits calcineurin activities in T cells, and thus exerts immunosuppressive effects. CsA also binds to cyclophilin D (CypD), which results in inhibition of mPTP opening and subsequent cell deaths^9^. In experimental animals, intravenous administered CsA decreases infarct size^10^. In clinical trials, however, intravenously administered CsA (2.0 mg/kg) failed to improve neurological outcomes after ischemic stroke^11^.This discrepancy raises at least two possibilities. First, monotherapy targeting only mPTP was insufficient in humans. Second, drug delivery to target organ was insufficient. In this study, we prepared mice lacking both CypD and CCR2 to elucidate whether targeting two mechanisms, i.e. mPTP opening and inflammation, confers substantial neuroprotection as evidenced by marked reduction in infarct size and recovery of neurological deficit after cerebral IR. To overcome the second possibility, we developed a drug delivery system (DDS) composed of poly-lactic/glycolic acid nanoparticles (PLGA-NPs) and incorporated CsA (CsA-NP)^12–16^. Nano-sized materials accumulate to sites with increased vascular permeability such as tissues/organs after IR^17,18^. Furthermore, PLGA-NPs are taken up by circulating monocytes and phagocytic cells in reticuloendothelial organs after intravenous administration^19–21^. HMG-CoA (3-hydroxy-3-methylglutaryl coenzyme-A) reductase inhibitor, statin, has inhibitory effects on monocyte-mediated inflammation independent of its lipid-lowering effect. We have developed PLGA-NPs incorporating pitavastatin (Pitava-NPs) and demonstrated their therapeutic effects in various cardiovascular diseases^12–16^.

Therefore, the aim of this study is to elucidate the simultaneous blockade of mPTP opening and monocyte-mediated inflammation could be an effective strategy for the treatment of cerebral IR injury. Recently, the revascularization after cerebral infarction is becoming a common procedure. This progress raises a possibility that drugs which could not show their efficacy in past clinical trials exert their therapeutic effects in the current era of early reperfusion therapy by combining with appropriate DDS. Hence, we have performed transient occlusion of middle cerebral artery to simulate cerebral ischemia and early reperfusion. In this model, we have tested an efficacy of simultaneous administration of CsA-NP and Pitava-NP on infarct size and neurological outcomes after cerebral IR.

## Results

### Double knockout of CypD and CCR2 ameliorated cerebral IR injury compared to CypD- or CCR2-single knockout in mice

**Figure 1:**
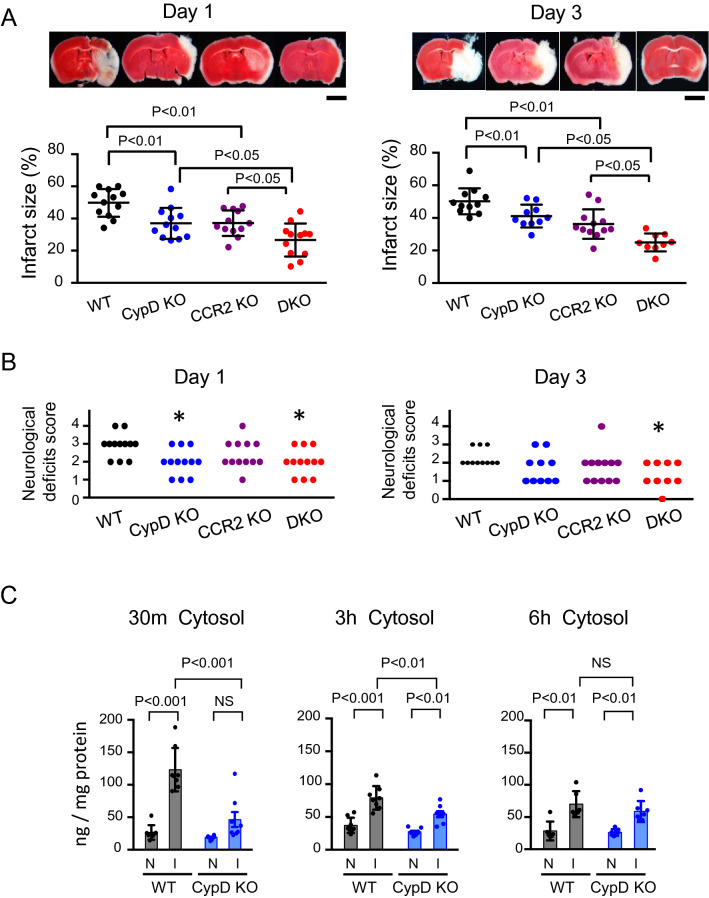
Double knockout of CypD and CCR2 decreased cerebral IR injury compared to CypD- or CCR2-single knockout in mice. (**A**) Cross-sectional pictures of brains harvested 1 day and 3 days after IR injury. Graphs demonstrate quantitative results of TTC-negative infarct area. The data are represented as the mean ± SD (N = 9–12 mice per group), and they were analyzed by one-way ANOVA followed by Bonferroni’s multiple comparison tests. Scale bar: 2 mm. (**B**) Neurological deficits score evaluated 1 day and 3 days after IR injury. Data were analyzed by the nonparametric Kruskal–Wallis test. *P < 0.05 versus WT mice. (**C**) Quantitative data of cytochrome c in the cytosol fraction isolated from brain tissues after IR injury. I: ischemic hemisphere, N: nonischemic hemisphere. The data represent the mean ± SD and compared using two-way ANOVA followed by Bonferroni’s multiple comparison tests.

Double-mutant mice that lack both CypD and CCR2 (double-knockout; DKO) were prepared to examine the effect of CypD-mediated mPTP opening and CCR2-mediated inflammation. As shown in Fig. 1A, deletion of CypD or CCR2 decreased the infarct size on day 1 and day 3, and double deletion of CCR2 and CypD further decreased the infarct size compared to CypD- or CCR2-single-knockout mice. The neurological deficit score was significantly reduced both in CypD-KO and DKO mice at day 1; however, a significant reduction of the score was observed only in DKO mice at day 3 (Fig. 1B), which suggests that the blockade of either CypD or CCR2 is insufficient, and simultaneous targeting of both CypD and CCR2, i.e. both mPTP opening and inflammation, is necessary to improve neurological outcomes after cerebral IR injury.

### Deletion of CypD inhibited the leakage of cytochrome c to the cytosol at early phase after cerebral IR injury

Cytochrome c is released from the mitochondria as a result of mPTP opening after cerebral IR injury. Hence, we quantified cytochrome c released to the cytosol after cerebral IR injury. As shown in Fig. 1C, cytochrome c in the cytosol increased in the brain tissue exposed to IR injury, but not in the non-ischemic brain, at 30 min after reperfusion, and this induction lasted for 6 h after reperfusion. In the CypD-KO mice, the concentration of cytochrome c in the cytosol of the ischemic brain was not significantly increased at 30 min after reperfusion. At 3 h after reperfusion, the cytochrome c concentration was also increased in the CypD-KO mice, however, its concentration of cytochrome c was significantly lower compared to that in the wild-type (WT) mice (Fig. 1C). These results indicate that the leakage of cytochrome c from the mitochondria is mainly regulated by CypD in the super acute phase after IR injury (30 min). In contrast, relative contribution of CypD decreased in a time-dependent manner.

### Deletion of CypD did not inhibit inflammation after cerebral IR injury

**Figure 2:**
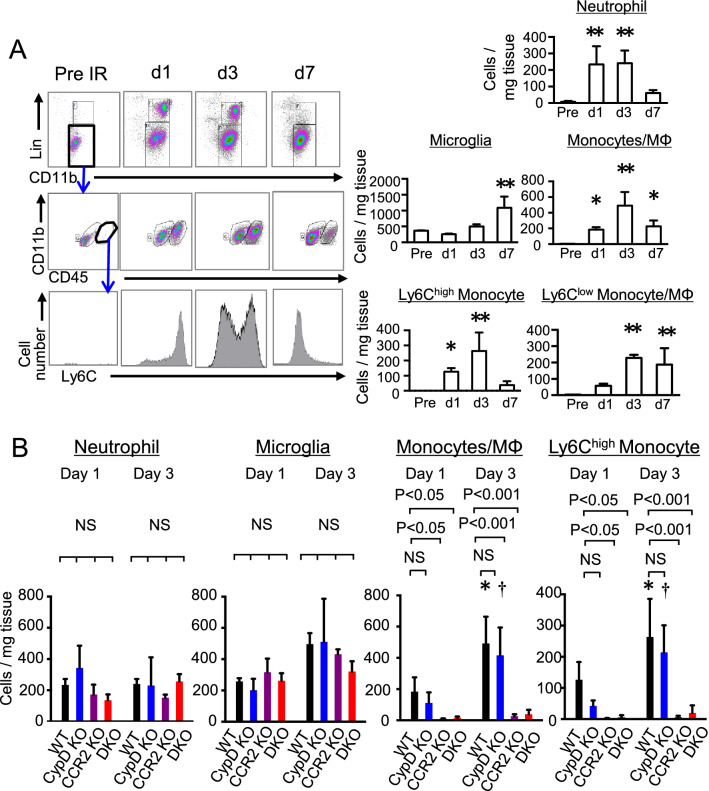
Recruitment of Ly6C^high^ monocytes was abrogated by CCR2-KO, but not by CypD-KO. (**A**) Accumulation of CD45^+^ cells (leukocytes and microglia) after cerebral IR injury. The number of cells were quantified in the hemispheres exposed to IR injury. The data represent the mean ± SD (N = 3–8). *P < 0.05, **P < 0.001 versus WT in preischemia. Statistical analyses were performed by one-way ANOVA with Dunnett’s tests. (**B**) Quantitative data showing the number of CD45^+^ cells in the cerebral tissues isolated from WT or CypD-KO mice after IR injury. The data represent the mean ± SD (N = 6–8). *P < 0.05 versus WT at day 1, ^†^*P* < 0.05 versus CypD-KO mice at day 1. Statistical analyses were performed by two-way ANOVA with Bonferroni’s multiple comparison tests.

To clarify the role of CypD-mediated mPTP opening in inflammation after cerebral IR injury, accumulation of inflammatory cells was evaluated by flow cytometry after transient occlusion of the middle cerebral artery (MCAO). In the CD45^+^ leukocytes isolated from the infarcted hemisphere that was exposed to IR injury, the number of microglia did not increase between day 1 and day 3 and gradually increased from day 3 to day 7. The number of neutrophils rapidly increased on day 1 and decreased from day 3 to day 7. Monocytes increased in the ischemic hemisphere from day 1 and reached their peak on day 3. The percentages of Ly6C^high^ monocytes were 67.5% (day 1), 50.2% (day 3) and 15.2% (day 7) (Fig. 2A). The number of microglia, neutrophils, total monocytes or Ly6C^high^ monocytes in the cerebral tissue was comparable between CypD-KO mice and WT mice after IR injury, despite the decrease in infarct size (Fig. 2B). On the other hand, the number of microglia and neutrophils was comparable in the CCR2-KO mice, but the deletion of CCR2 abrogated the recruitment of inflammatory monocytes on day 1 and day 3 (Fig. 2B).

**Figure 3:**
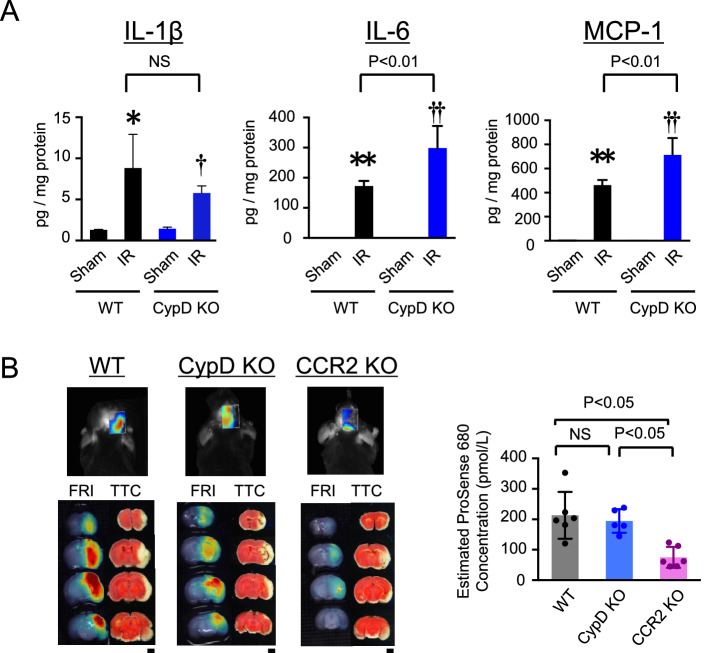
Depletion of CypD did not inhibit inflammation after cerebral IR injury. (**A**) Protein level of inflammatory cytokines in homogenized cerebral tissues at 24 h after reperfusion. Data are presented as the mean ± SD (N = 4 mice per sham group, N = 8 mice per IR group). *P < 0.01 versus sham in WT mice, **P < 0.001 versus sham in WT mice, ^†^*P* < 0.05 versus sham in CypD-KO mice, ^††^*P* < 0.001 versus sham in CypD-KO mice. Statistical analyses were performed by two-way ANOVA with Bonferroni’s multiple comparison tests. (**B**) Representative FMT (above) and FRI (below) images demonstrating protease activities at 72 h after IR visualized by ProSense 680. The graph indicates ProSense 680 concentrations in the cerebral tissues as estimated from the fluorescent intensity. The data represent the mean ± SD (N = 5–6 mice per group), and they were analyzed by one-way ANOVA followed by Bonferroni’s multiple comparison tests. FMT: fluorescence molecular tomography. FRI: fluorescence reflectance imaging.

Furthermore, the protein levels of inflammatory cytokines including monocyte chemoattractant protein-1 (MCP-1) and Interleukin-6 (IL-6) were increased in the homogenized brain tissues obtained from the CypD-KO mice (Fig. 3A). In contrast, siRNA-mediated knockdown of CypD in RAW264.7 cells demonstrated no significant effects on expression of MCP-1 and IL-6 mRNA (Supplemental Fig. 1). Fluorescence molecular tomography (FMT) imaging using ProSense-680 demonstrated increased protease activities around the TTC (2,3,5-triphenyl-2H-tetrazolium chloride)-negative infarcted area, showing enhanced inflammatory milieu in the penumbra. Consistent with flow cytometric analysis, the protease activities were significantly decreased in the CCR2-KO mice but not in the CypD-KO mice (Fig. 3B), indicating that the inhibition of CypD does not ameliorate acute inflammation after cerebral IR injury, and inflammation could be a distinct therapeutic target in addition to CypD-mediated mPTP opening, and that simultaneous targeting of both mPTP opening and inflammation could be an effective combination therapy against cerebral IR injury.

### In vivo distribution of PLGA-NPs after cerebral IR injury

**Figure 4:**
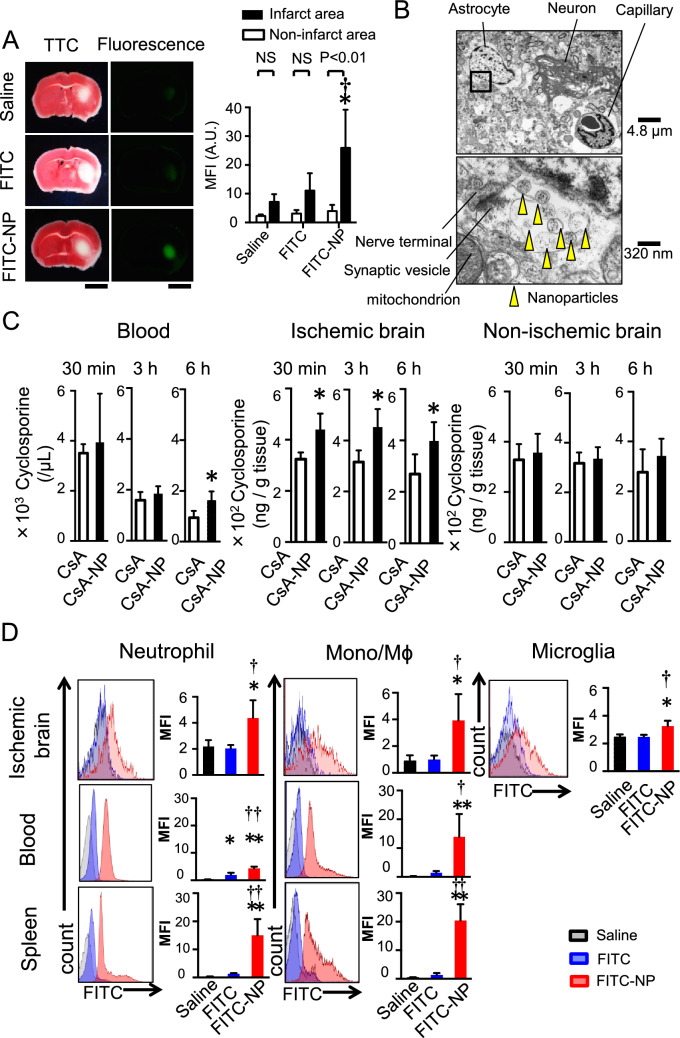
In vivo distribution of PLGA-NPs after cerebral IR injury. (**A**) Representative light (left) and fluorescent (right) photographs of cross-sectioned brains 6 h after an intravenous injection of saline, FITC or FITC-NPs. In the light images, the brains were stained with TTC to determine the infarct area. Scale bar: 2 mm. Data are presented as the mean ± SD (N = 4 mice per group). *P < 0.01 versus saline in the infarct area, ^†^*P* < 0.05 versus FITC in the infarct area according to two-way ANOVA followed by Bonferroni’s multiple comparison tests. (**B**) Electron microscopic images of ischemic brain tissue 6 h after an intravenous injection of FITC-NPs. Yellow arrowheads indicate nanoparticles. (**C**) Blood and tissue (ischemic hemisphere, nonischemic hemisphere) concentrations of CsA. Data are presented as the mean ± SD (N = 8 mice per group). *P < 0.05 versus CsA calculated by unpaired *t*-tests. (**D**) Flow cytometric analysis of leukocytes isolated from the brain, blood and spleen. Histograms indicate representative data showing the distribution of FITC fluorescence, and bar graphs show quantitative results. MFI: mean fluorescent intensity. Data are presented as the mean ± SD (N = 4–5 mice per group). *P < 0.05 versus saline, **P < 0.01 versus saline, ^†^*P* < 0.05 versus FITC, ^††^*P* < 0.01 versus FITC according to one-way ANOVA followed by Bonferroni’s multiple comparison tests.

To investigate the in vivo distribution of nanoparticles after cerebral IR injury, fluorescein-isothiocyanate (FITC) solution or FITC-NPs were intravenously injected at the time of reperfusion, and fluorescent signals were observed 6 h later. FITC signals were exclusively detected in the infarct area of the FITC-NP group, whereas faint FITC signals were observed in the brain from the FITC-solution and saline groups (Fig. 4A). The fluorescent intensity was less in the non-infarct area compared to the infarct area (Fig. 4A). Transmission electron microscopy revealed that PLGA-NPs were located in the extracellular matrix, phagocytes and endothelial cells of the capillaries in the ischemic brain parenchyma 6 h after reperfusion (Fig. 4B, Supplemental Fig. 2).

We examined CsA pharmacokinetics in mice after transient MCAO. CsA solution or CsA-NPs were intravenously injected at the time of reperfusion, and tissue concentration was measured. In the cerebral tissues exposed to IR injury, the tissue concentration of CsA was higher in the CsA-NP group than the CsA-solution group at 30 min after administration, which lasted for 6 h. In contrast, there were no significant differences in the tissue concentration of CsA between the CsA-NP group and the CsA-solution group in the nonischemic brain tissues (Fig. 4C). Nanoparticles are taken up by circulating monocytes and phagocytic cells in reticuloendothelial organs after intravenous administration; therefore, we examined FITC signals in the leukocytes of the brain, blood and spleen 24 h after reperfusion by flow cytometry. As shown in Fig. 4D, fluorescent signals were detected in the microglia, neutrophils and monocytes that were isolated from the brain after IR injury. Among leukocytes isolated from the peripheral blood and spleen, fluorescent signals were also detected in neutrophils and monocytes. These data indicate that PLGA-NPs were delivered to the ischemic brain parenchyma where the blood brain barrier (BBB) was disrupted in the early phase after cerebral IR and to leukocytes, especially monocytes, in the peripheral blood, spleen and the brain.

### CsA-NP reduced infarct size after cerebral IR injury by blocking mPTP opening

**Figure 5:**
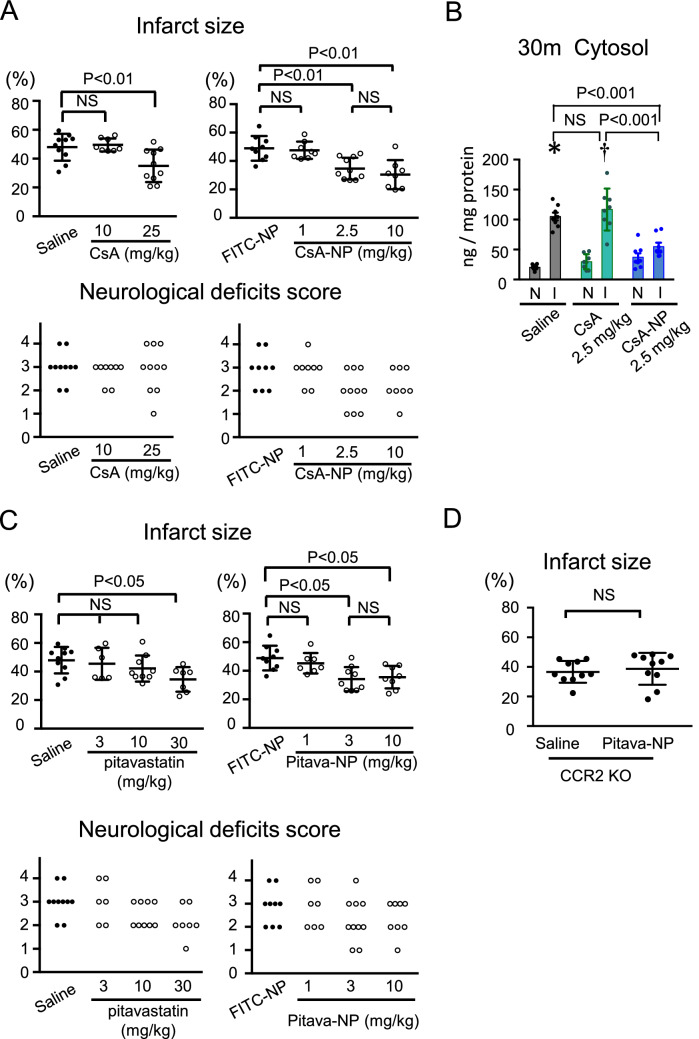
CsA-NP reduced infarct size after cerebral IR injury by blocking mPTP opening and Pitava-NP reduced infarct size after cerebral IR injury by blocking CCR2-mediated inflammation. (**A**) Quantitative data of infarct size and neurological deficits score 24 h after IR. The data of infarct size represent the mean ± SD (N = 8–12 mice per group), and they were analyzed by one-way ANOVA followed by Bonferroni’s multiple comparison tests. Data of neurological deficits score were analyzed by the nonparametric Kruskal–Wallis test. (**B**) Quantitative data of cytochrome c in the cytosol fraction 30 min after reperfusion. The data represent the mean ± SD. *P < 0.001 versus saline in the non-ischemic hemisphere, ^†^*P* < 0.001 versus CsA in the noninfarct hemisphere, as analyzed by two-way ANOVA followed by Bonferroni’s multiple comparison tests. I: ischemic hemisphere, N: non-ischemic hemisphere. (**C**) Quantitative data of infarct size and neurological deficits score 24 h after IR injury. The data of infarct size represent the mean ± SD (N = 6–10), and they were analyzed by one-way ANOVA followed by Bonferroni’s multiple comparison tests. The data of neurological deficits score were analyzed by the nonparametric Kruskal–Wallis test. (**D**) The effects of Pitava-NP on the infarct size in CCR2-KO mice. The data represent the mean ± SD (N = 10 per group), and they were compared using unpaired *t* tests.

The results of in vivo trace experiments prompted us to examine the therapeutic effect of CsA-incorporating nanoparticles on the infarct size after cerebral IR injury. In mice, after transient MCAO, intravenous administration of CsA solution at the time of reperfusion reduced the infarct size, but the reduction was only observed when a high dose (25 mg/kg) CsA was administered (Fig. 5A). In contrast, CsA-NP containing 2.5 mg/kg CsA reduced the infarct size. Further reduction of the infarct size, however, was not observed in mice treated with CsA-NP containing 10 mg/kg CsA (Fig. 5A). In terms of neurological outcome, CsA-NP did not significantly decrease the neurological deficit score compared to FITC-NPs (Fig. 5A). We examined the effects of CsA and CsA-NP on the cytochrome c leakage. CsA at 2.5 mg/kg did not decrease the cytochrome c concentration in the cytosol, whereas 2.5 mg/kg CsA-NP, which decreased the infarct size, attenuated the cytochrome c leakage (Fig. 5B), suggesting that the primary mechanism that underlies CsA-NP-induced neuroprotection against IR injury is mediated through the inhibition of mPTP opening.

### Pitava-NP reduced infarct size after cerebral IR injury by blocking CCR2-mediated inflammation

Accumulating data indicate beneficial effects of statins on inflammation, which are independent of their lipid-lowering effect^28,29^. We previously reported that Pitava-NP inhibited recruitment of Ly6C^high^ monocytes to the heart after IR injury or permanent occlusion of the coronary artery in mice^14,19^. In atherosclerotic plaques, Pitava-NP decreased the accumulation of macrophages within the plaque^18^. Therefore, we decided to use Pitava-NP to suppress inflammation after cerebral IR injury. In mice subjected to 60 min of MCAO and 24 h of reperfusion, intravenously administration of pitavastatin (30 mg/kg) at the time of reperfusion decreased the infarct size. Similarly, Pitava-NP containing 3.0 mg/kg pitavastatin decreased the infarct size after IR. (Fig. 5C). In contrast, Pitava-NP did not improve the neurological symptoms. (Fig. 5C) To determine the detailed molecular mechanisms, we examined the therapeutic effects of Pitava-NP containing 3.0 mg/kg pitavastatin in CCR2-KO mice. As shown in Fig. 5D, Pitava-NP did not decrease the infarct size in CCR2-KO mice, suggesting that Pitava-NP exerted its therapeutic efficacy via CCR2-mediated inflammation.

### Combination of CsA-NP and Pitava-NP enhanced neuroprotection

**Figure 6:**
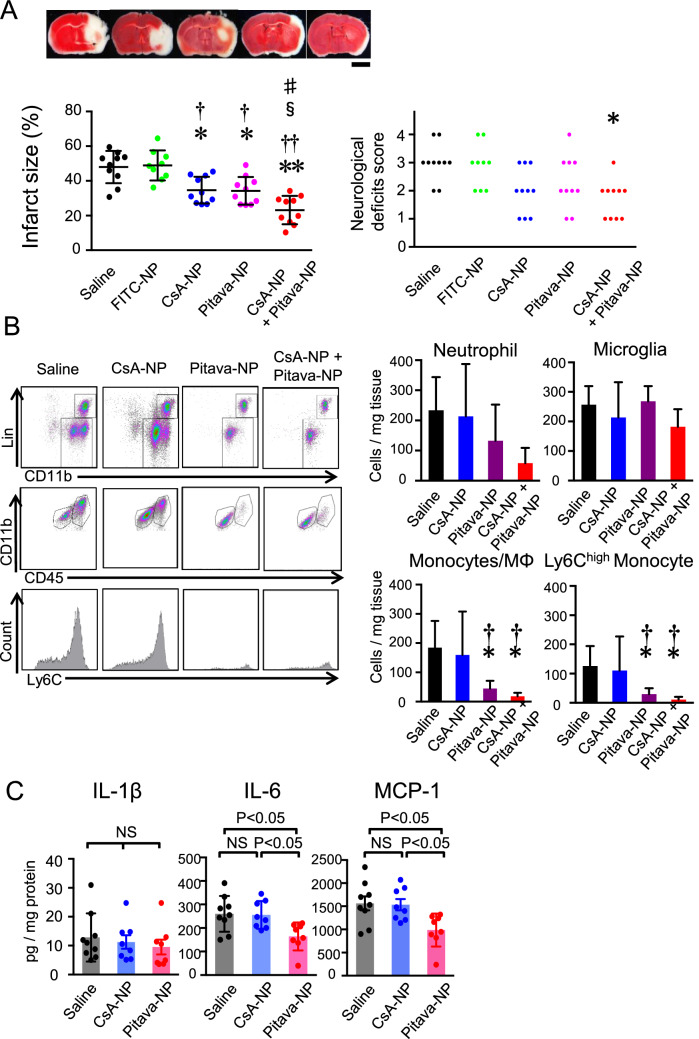
Combination therapy with CsA-NP and Pitava-NP enhanced neuroprotection against cerebral IR injury. (**A**) Infarct size and the neurological deficits score at 24 h after IR injury. CsA-NP contains 2.5 mg/kg CsA, and Pitava-NP contains 2.5 mg/kg pitavastatin. The left graph presents the quantitative results of the infarct size presented as the mean ± SD (N = 9–12 mice per group). These data were analyzed by one-way ANOVA followed by Bonferroni’s multiple comparison tests. The right graph shows the results of the neurological deficits score. These data were analyzed by the nonparametric Kruskal–Wallis test. *P < 0.01 versus saline, **P < 0.001 versus saline, ^†^*P* < 0.01 versus FITC-NP, ^††^*P* < 0.001 versus FITC-NP, ^§^*P* < 0.05 versus CsA-NP, ^#^*P* < 0.05 versus Pitava-NP. (**B**) Flow cytometric analysis of the CD45^+^ cells isolated from cerebral tissues after IR. The data represent the mean ± SD (N = 6–8), and they were analyzed by one-way ANOVA followed by Bonferroni’s multiple comparison tests. *P < 0.05 versus saline, ^†^*P* < 0.05 versus CsA-NP. (**C**) Protein level of inflammatory cytokines in the homogenized cerebral tissues measured 24 h after reperfusion. Data are presented as the mean ± SD (N = 8–9 mice per group), and they were compared using one-way ANOVA followed by Bonferroni’s multiple comparison tests.

The infarct size and neurological deficit score were evaluated in mice treated with FITC-NP (used as a control for nanoparticles), CsA-NP (2.5 mg/kg), Pitava-NP (3.0 mg/kg) or a cocktail containing CsA-NP (2.5 mg/kg) and Pitava-NP (3.0 mg/kg) at the time of reperfusion. CsA-NP and Pitava-NP showed a significant reduction of infarct size at 24 h after reperfusion compared to saline and FITC-NP (Fig. 6A). Importantly, simultaneous administration of CsA-NP and Pitava-NP further reduced the infarct size and ameliorated the neurological deficit score compared to single administration of CsA-NP or Pitava-NP (Fig. 6A). To elucidate the effects of Pitava-NP on inflammation after cerebral IR injury, we performed flow cytometric analysis and found that Pitava-NP decreased the recruitment of inflammatory monocytes to the ischemic brain, whereas CsA-NP did not (Fig. 6B). Similarly, the protein levels of MCP-1 and IL-6 in the infarcted hemisphere were significantly reduced by Pitava-NP and but not by CsA-NP, compared to that in the saline group (Fig. 6C).

## Discussion

Opening of mPTP and inflammation are two major mechanisms of IR injury. CypD regulates the opening of mPTP, which induces neuronal cell death immediately after reperfusion^22,23^. Reactive oxygen species (ROS), calcium overload and rapid pH correction, any of which are observed after cerebral IR, induce the opening of mPTP, and cytochrome c released from the mitochondrial matrix induces cell death^2^. However, the role of mPTP opening in the subacute phase, when inflammatory cells accumulate, has not been clarified. Therefore, we prepared double-mutant mice that lack both CypD and CCR2 to examine relative importance of mPTP opening and inflammation in the disease progress.

The quantitative data of cytosol cytochrome c suggests that the CypD-mediated mPTP opening triggers neuronal cell death only in the early phase after reperfusion. In the present study, on the other hand, deletion of CypD did not suppress inflammation in cerebral tissues after IR despite the reduction of infarct size. One possible reason of this discrepancy is that the deletion of CypD decreased neural cell deaths, and survived cells exacerbated inflammation by releasing inflammatory cytokines. Another possible reason is explained by Signal Transducers and Activator of Transcription-3 (STAT3). STAT3 in the mitochondrial matrix binds to CypD and inhibits mitochondrial ROS production^32^. In CypD-KO mice, this inhibitory effect of STAT3 on ROS production might be abrogated by the absence of CypD. In addition, there is a possibility that deletion of CypD suppressed mPTP opening and subsequent cell death in monocytes/macrophages, which in turn caused prolonged inflammation accompanied with increased cytokine expressions. Nonetheless, in vivo results suggest that it is insufficient to target only CypD during the early phase after IR injury.

The role of CCR2-mediated inflammation after cerebral IR injury has been demonstrated in experimental animals^3,24^; however, contradicting results that a selective CCR2 antagonist exacerbated the IR injury have been also reported^25^. Resent reports have shown that CCR2-dependent recruitment of monocytes/macrophages is a double-edge sword; they exacerbate acute brain injury but, on the other hand, promote the recovery of neurological functions because inflammatory macrophages recruited by a CCR2-dependent mechanism differentiate into reparative macrophages in the later phase after IR injury^26^. In this study, deletion of CCR2 decreased the infarct size 24 and 72 h after reperfusion accompanied with decreased accumulation of inflammatory monocytes in the injured brain. Furthermore, the additional knockdown of CCR2 in CypD single knockout, i.e. double knockout of CypD and CCR2, decreased the infarct size and improved neurological deficit score at 24 and 72 h after IR, suggesting that inflammation is a distinct therapeutic target in addition to CypD-mediated mPTP opening, which may contribute to neurological outcome. We could not completely exclude a possibility that the collateral blood flow in CypD-KO and CCR2-KO mice may be better than that of WT mice because infarct volumes, as assessed by TTC staining, were significantly smaller in these KO mice in as early as 24 h; however, there were no significant differences in cerebral blood flow during transient MCAO between WT mice and these KO mice. In addition, the significant reduction of infarct size in CCR2-KO mice before the peak of monocyte/macrophage accumulation, raises a possibility that the genetic manipulation may also influence the ischemic tolerance of neural cells^27^.

We used cyclophilin inhibitor CsA to inhibit cell death induced by mPTP opening and prepared polymeric nanoparticles incorporating CsA to enhance the drug delivery to the ischemic cerebral tissues. In vivo trace experiments indicated enhanced drug delivery to the ischemic cerebral tissues by PLGA-NPs. Fluorescent signals emitted by FITC-NP were mainly observed in the infarct lesion, suggesting that the nanoparticles were delivered to the lesion where BBB was disrupted. The tissue concentration of CsA, as demonstrated in Fig. 4C, also indicates the selective delivery of CsA to the ischemic cerebral tissues. This point is an advantage of PLGA-NPs because undesired adverse effects that may occur in intact cerebral tissues could be avoided.

To inhibit inflammation, we have selected an HMG-CoA reductase inhibitor, pitavastatin. Pitavastatin has several advantages when considering clinical application. First, it is already clinically available. Second, it exhibits strong anti-inflammatory and vasoprotective effects compared to other statins^28^. We have previously reported that Pitava-NP suppresses MCP-1-induced macrophage chemotaxis (in vitro)^15^ and macrophage accumulation in hearts exposed to IR (in vivo)^16^. Thus, we have selected pitavastatin among several statins in this study. The combined administration of CsA-NPs and Pitava-NPs demonstrates that simultaneous targeting of mPTP opening and inflammation is an effective and favorable combination that covers two major mechanisms of IR injury. Accumulating evidence, however, indicates that accumulation of macrophages to infarct tissues are important for clearance of myelin debris and functional recovery after cerebral infarction^29,30^. Therefore, further research is needed to elucidate the effects of Pitava-NPs on accumulation and function of reparative macrophages in later phase after reperfusion. In addition, optimization of the administration protocol of Pitava-NP is important for clinical translation. It is important to suppress acute phase inflammation, while accumulation of reparative macrophages should not be too much suppressed.

Nanotechnology-based DDS (nano-DDS) is a promising tool for effective delivery of drugs, and various forms of DDS have been developed, some of which are already clinically available or tested in clinical trials. The advantages of our nano-DDS using PLGA-NPs include the fact that PLGA has been already approved by the US Food and Drug Administration, the European Medicine Agency (EMA) and the Japanese regulatory agency (PMDA) for clinical use. We have already completed clinical trials that tested the safety and efficacy of Pitava-NPs: a phase I/IIa clinical trial (UMIN000008011) in patients with critical limb ischemia (intramuscular injection) and a phase I clinical trial that tested the safety of intravenously administered Pitava-NPs (UMIN000014940, UMIN000019189)(single and repeated injections)^31^. In addition, a phase II clinical trial in patient with pulmonary arterial hypertension is ongoing (UMIN000032531). In either of these clinical trials, no adverse events have been reported that have causal relationships with Pitava-NP. Furthermore, CsA has been used for many years as an immunosuppressant in patients after organ transplantation. These points indicate that our strategy of using CsA-NP and Pitava-NP has an advantage that clinical application is feasible and could be achieved in a relatively short time compared with de novo synthesized drugs.

In conclusion, simultaneous targeting of mPTP and monocyte mediated-inflammation can be an effective and appropriate strategy for protection of the brain from IR injury. Nanoparticle-mediated delivery of CsA and pitavastatin to the cerebral tissue could be a novel and clinically feasible therapy for protecting the brain from IR injury, a major clinical problem with no medical solutions.

## Methods

### Experimental animals

All animal experimental protocols were reviewed and approved by the committee on the Ethics of Animal Experiments, Kyushu University Faculty of Medicine, and conducted in accordance with the American Physiological Society guidelines. Male wild-type (WT) mice (C57Bl/6 J background) were purchased from CLEA Japan, Inc. (Tokyo, JAPAN), and CypD-knockout (KO) mice were purchased from Jackson Laboratories (Stock#: 009,071, Bar Harbor, ME). CCR2-KO mice (C57Bl/6 J and 129/svjae hybrids, provided by Dr. Charo)^32,33^ backcrossed with C57Bl/6 J mice at least 10 times were used for experiments. The animals were maintained under a 12-h light–dark cycle with free access to normal chow and water.

### Transient middle cerebral artery occlusion

Male mice aged 8–12 weeks and weighing 20–30 g were used for experiments. Mice were anesthetized using isoflurane (1.5%) during surgery, and body temperatures were maintained at 35–37 °C with a surface heating pad during the entire procedure. Transient brain ischemia was induced as described previously^34^. A midline incision of the neck skin was performed to expose the right common carotid artery. Next, a silicone-coated 6–0 monofilament (Doccol Corp, Redlands, CA) was inserted from the right external carotid artery to the origin of the middle cerebral artery (MCA). After 60 min of occlusion of MCA (MCAO), the cerebral blood flow was restored by removing the filament. The regional cerebral blood flow was monitored using laser Doppler flowmetry (OMEGAWAVE, INC., Tokyo, Japan), and mice were included only when the blood flow was reduced to less than 20% compared to the preischemic baseline level during ischemia and was restored to 80% or more compared to the preischemic level within 10 min after reperfusion. For the sham group, surgical procedures except monofilament insertion were conducted.

### Neurobehavioral evaluation

The neurological performances were evaluated and graded using a modified Bederson score^35,36^. Each grading scale (0–4) indicates following conditions: 0, no neurological deficit (normal); 1, forelimb flexion (mild); 2, decreased resistance to lateral push towards the paretic side without circling (mild to moderate); 3, circling (moderate); 4, no spontaneous movement (severe).

### Assessment of infarct size

The brain was excised and sliced into 7 sequential cross-sections with 1.0 mm thickness. The sections were incubated with 2% 2,3,5-triphenyl-2H-tetrazolium chloride (TTC, Sigma Aldrich) in saline for 20 min at 37 °C, fixed in 10% neutral-buffered formalin for 24 h, and photographed with a stereomicroscope (HC-2500, Nikon). To avoid the influence of cerebral edema, the ImageJ software (version 1.46) was used to calculate the percent infarct size as [V_C_ − V_L_/V_C_] × 100, where V_C_ is the sum of all areas of control hemisphere and V_L_ is the sum of noninfarct areas in the lesion ipsilateral hemisphere. We have reused the same control mice in Fig. 5A,C and Fig. 6A, the same mice with CsA-NP 2.5 mg/kg in Fig. 5A and Fig. 6A and the same mice with Pitava-NP 3.0 mg/kg in Fig. 5C and Fig. 6A.

### Preparation of PLGA nanoparticles

PLGA with an average molecular weight of 20,000 Da and a copolymer ratio of lactide to glycolide of 75:25 (Wako Pure Chemical Industries, Osaka, Japan) was used as a matrix of nanoparticles, and polyvinyl alcohol (PVA-403; Kuraray, Osaka, Japan) was used as a dispersing agent. PLGA-NPs incorporating the fluorescent marker fluorescein-isothiocyanate (FITC; Dojin Chemical, Tokyo, Japan) (FITC-NP), CsA (Sigma Aldrich, MO) (CsA-NP) or pitavastatin (Kowa Pharmaceutical, Tokyo, Japan) (Pitava-NP) were prepared by the emulsion solvent diffusion method in purified water, as previously described^12,13^. FITC-NP contained 4.05% (wt/vol) FITC, the CsA-NP contained 2.45% (wt/vol) CsA, and the Pitava-NP contained 12.0% (wt/vol) pitavastatin. The average diameters of FITC-NP, CsA-NP and Pitava-NP were 223 nm, 188 nm and 159 nm, respectively. The surface charge (zeta potential) was also analyzed using a Zetasizer Nano system (Sysmex, Hyogo, Japan), which revealed that the nanoparticles were anionic [−24.8 mV (FITC-NP), −16.5 mV (CsA-NP) and −4.1 mV (Pitava-NP)].

### Transmission electron microscopy

The animals were intravenously injected with FITC-NPs at the time of reperfusion and euthanized 6 h later by intravenous injection of 100 mg/kg pentobarbital. Next, the mice were perfused with 30 mL of saline and 30 mL of 50% Karnofsky buffer. The brain samples were obtained from the ischemic hemisphere, and the sliced samples were fixed overnight. These small blocks of brain samples were dehydrated using a graded ethanol series. Next, ultrathin sections with 80 nm thickness were prepared with a diamond knife on an ultramicrotome (LEICA EM UC7, Wetzlar, Germany). The sections were collected on 150 mesh copper grids and stained with uranyl acetate and lead citrate. The specimens were observed using an H-7500 transmission electron microscope (Hitachi, Tokyo, Japan) with 80 kV accelerating voltage.

### Measurement of cyclosporine concentrations in plasma and tissues

The mice were subjected to 60 min of ischemia followed by reperfusion and were intravenously injected with 10 mg/kg CsA or CsA-NPs containing 10 mg/kg CsA at the time of reperfusion. Blood samples were collected in tubes with EDTA-2 K (Sigma-Aldrich). The brain samples were harvested at the indicated time points. Each sample was weighed and homogenized after separating into ischemic and nonischemic hemispheres. The CsA concentrations in the whole blood and cerebral tissue homogenates were measured by electro chemiluminescence immunoassay (ECLIA), which highly correlates with a validated HPLC method.

### Distribution of FITC-NP

The distribution of FITC-NP in the brain, peripheral blood leukocytes and spleen was examined. After cerebral IR, the animals were euthanized, and peripheral blood was drawn by cardiac puncture, with EDTA (ethylenediaminetetraacetic acid) serving as an anticoagulant. The red blood cells were lysed with VersaLyse Lysing Solution (Becton Dickinson Bioscience, Inc., CA). The spleen was harvested and triturated in HBSS (Hank’s Balanced Salt Solution) on ice and filtered through a 70-µm cell strainer. The distribution of FITC-NPs in the peripheral blood and spleen was analyzed with a Gallios Flow Cytometer (Beckman Coulter, Inc, CA).

### Mitochondria isolation

The mouse brain mitochondria were isolated using a mitochondria isolation kit (Abcam, MA) according to the manufacturer’s protocol. Briefly, the brain was quickly excised, minced on ice, resuspended in 1 mL of washing buffer, and homogenized using a glass Dounce homogenizer. The homogenate was centrifuged at 1,000 × g and 4 °C for 10 min. The supernatant was recentrifuged at 12,000×*g* for 15 min to pellet the mitochondria, and the resultant supernatant was used as the supernatant fraction. The pellet was washed twice with isolation buffer.

### Flow cytometry

Peripheral blood was drawn by cardiac puncture, and the red blood cells were lysed with VersaLyse Lysing Solution (Becton Dickinson Bioscience, Inc., CA, USA) for 10 min at room temperature. The brains were excised and digested with a cocktail of 125 U/ml collagenase type IV, 60 U/ml DNAase I and 60 U/ml hyaluronidase (all enzymes were obtained from Sigma-Aldrich) in PBS containing 20 mM HEPES at 37 °C for 30 min in a shaker. Mononuclear cells were isolated by 37/70% Percoll (GE Healthcare) density gradient centrifugation. The spleen was harvested and triturated in HBSS (Hank’s Balanced Salt Solution) on ice and filtered through a 70 µm cell strainer. After blocking the Fc receptor with an anti-CD16/32 monoclonal antibody (BD Pharmingen, San Diego, CA) for 5 min at 4 °C, cell suspensions were incubated with a cocktail of monoclonal antibodies against leukocytes (CD45-PE-Cy7, 30-F11), myeloid cells (CD11b-APC or APC-Cy7, M1/70) and monocyte subsets (Ly6C-FITC or PerCP-Cy5.5, AL-21) for 1 h at 4 °C. A cocktail of monoclonal antibodies against T cells (CD90-PE, 53–2.1), B cells (B220-PE, RA3-6B2), natural killer (NK) cells (CD49b-PE, DX5 and NK1.1-PE, PK136) and granulocytes (Ly6G-PE, 1A8) was used as a lineage (Lin) marker. All leukocytes were subsequently analyzed with a Gallios Flow Cytometer. The microglia were identified as CD45^intermediate^CD11b^intermediate^Lin^low^Ly6C^low^ cells. The monocytes and neutrophils were identified as CD45^high^CD11b^high^Lin^low^Ly6C^high/low^ and CD45^high^CD11b^high^Lin^high^ cells, respectively. The lymphocytes were identified as CD45^high^CD11b^low^Lin^high^ cells. (Supplemental Fig. 3).

### Enzyme-linked immunosorbent assay (ELISA)

The brain was excised, minced on ice, and homogenized using a glass Dounce homogenizer with lysis buffer (50 mM Tris–HCl (pH 7.5), 150 mM NaCl, 1% NP40, 0.1% SDS, 0.5% deoxycholic acid, 10 mM Na_4_P_2_O_7,_ 5 mM EDTA, 0.1 mM Na_3_VO_4_, 1 mM NaF, and protease inhibitor cocktail). The lysates were centrifuged at 4 °C for 10 min, and the supernatants were collected. Interleukin-1β (IL-1β), IL-6 and monocyte chemoattractant protein-1 (MCP-1) concentrations were measured with commercially available ELISA kits (R&D Systems, Minneapolis, Minnesota) according to the manufacturer’s instructions.

### Fluorescence molecular tomography (FMT) and fluorescence reflectance imaging (FRI)

The near-infrared fluorescence imaging probe ProSense 680 (PerkinElmer, Inc., Waltham, MA) was used for the visualization of protease activities including cathepsin B, L, S and plasmin. In the mouse IR model, 5 nmol of ProSense 680 (Ex/Em = 680/700 nm) was intravenously administered 48 h after reperfusion. Mice were anesthetized 72 h later with isoflurane (1.5%) inhalation and scanned with the FMT-2000 system (PerkinElmer, Inc., Waltham, MA). Next, the mice were euthanized, and the brains were harvested. The excised brains were cut into sequential cross sections of 2.0 mm thickness, stained with TTC and imaged by FRI by using the planar imaging capability of FMT-2000.

### Cell culture

RAW264.7 cells were cultured with RPMI 1,640 medium (Lonza, Basel, Switzerland) containing 10% fetal bovine serum (FBS) (Sigma-Aldrich, St. Louis, MO) and 1% penicillin/streptomycin in an incubator set to 37 °C and 5% CO_2_. Lipopolysaccharide (LPS) (Sigma‐Aldrich) and mouse interferon-γ (Roche, Basel, Switzerland) were used for stimulations. For loss-of function experiments, CypD siRNA (Dharmacon, Lafayette, CO) or control non-targeting siRNA was transfected with SilenceMag (OZ Biosciences, Marseille, France). Culture supernatant was exchanged 24 h later and used for experiments.

### RNA extraction and semi-quantitative real-time PCR

RNA was extracted with illustra RNAspin Mini Kit (GE Healthcare Bioscience, Pittsburgh, PA) according to the manufacture’s instruction. cDNA was prepared with PrimeScript RT-PCR Kit (TAKARA BIO INC, Shiga, Japan). Semi-quantitative real-time PCR was performed with SYBR Premix DimerEraser (TAKARA BIO Inc.) and a StepOnePlus real-time PCR system (Applied Biosystems, Waltham, MA). The primer sequences are as follows: CypD-Fw 5′-CGACTTCACCAACCACAATG-3′ and CypD-Rv 5′-ACATCCATGCCCTCTTTGAC-3′, Il1-b-Fw 5′-GCCCATCCTCTGTGACTCAT-3′, and Il-1b-Rv 5′-AGGCCACAGGTATTTTGTCG-3′, Mcp-1-Fw 5′-AGGTCCCTGTCATGCTTCTG-3′ and Mcp-1-Rv 5′-TCTGGACCCATTCCTTCTTG-3′, TNF-α-Fw 5′-AGCCCCCAGTCTGTATCCTT-3′ and TNF-α-Rv 5′- CTCCCTTTGCAGAACTCAGG-3′, iNos-Fw 5′-TGAGGCTGAAATCCCAGCAG-3′ and iNos-Rv 5′-CTCTGAGGGCTGACACAAGG-3′, Il-6-Fw 5′-CCACTTCACAAGTCGGAGGC-3′ and Il-6-Rv 5′-GCAAGTGCATCATCGTTGTTCATAC-3′, β-actin-Fw 5′-CCTGAGCGCAAGTACTCTGTGT-3′ and β-actin-Rv 5′-GCTGATCCACATCTGCTGGAA-3′. Data were calculated by the ΔΔCt method and expressed in arbitrary units that were normalized by β-actin.

### Statistical analyses

Data are expressed as the mean ± standard deviation (SD). Statistical analyses of the differences between two groups were performed using the unpaired t-test. Analyses of the differences among three or more groups were performed using one-way or two-way ANOVA, followed by Bonferroni’s or Dunnett’s post hoc multiple comparison tests. Analysis of the neurological deficits score was performed by the nonparametric Kruskal–Wallis test, followed by Bonferroni’s post hoc multiple comparison tests. All statistical analyses were performed with the Prism software version 6.0 (Graph Pad Software, San Diego, California, USA), and P values less than 0.05 were considered to be significant.

## Supplementary information

Supplementary file1
