# Supplementary material for: Simultaneous targeting of mitochondria and monocytes enhances neuroprotection against ischemia–reperfusion injury
Source: Sci Rep. 2020 Sep 2;10:14435. doi: 10.1038/s41598-020-71326-x (PMC7468234; doi:10.1038/s41598-020-71326-x)
Supplement: Supplementary file 1 — Supplementary file1 [file 41598_2020_71326_MOESM1_ESM.pdf]

Simultaneous Targeting of Mitochondria and Monocytes Enhances Neuroprotection against Ischemia-reperfusion Injury

Arihide Okahara[1], Jun-ichiro Koga[1,2*], Tetsuya Matoba[1], Masaki Fujiwara[1], Masaki Tokutome[1], Gentaro Ikeda[1], Kaku Nakano[2], Masaki Tachibana[3], Tetsuro Ago[3], Takanari Kitazono[3], Hiroyuki Tsutsui[1], Kensuke Egashira[2,4]

[1]Department of Cardiovascular Medicine, Graduate School of Medical Sciences, Kyushu University, Fukuoka, Japan; [2]Department of Cardiovascular Research, Development, and Translational Medicine, Center for Cardiovascular Disruptive Innovation, Kyushu University, Fukuoka, Japan; [3]Department of Medicine and Clinical Science, Graduate School of Medical Sciences, Kyushu University, Fukuoka, Japan; [4]Department of Translational Medicine, Kyushu University Graduate School of Pharmaceutical Sciences, Fukuoka, Japan.

* Correspondence author: Jun-ichiro Koga, MD, PhD;
E-mail: j-koga@cardiol.med.kyushu-u.ac.jp

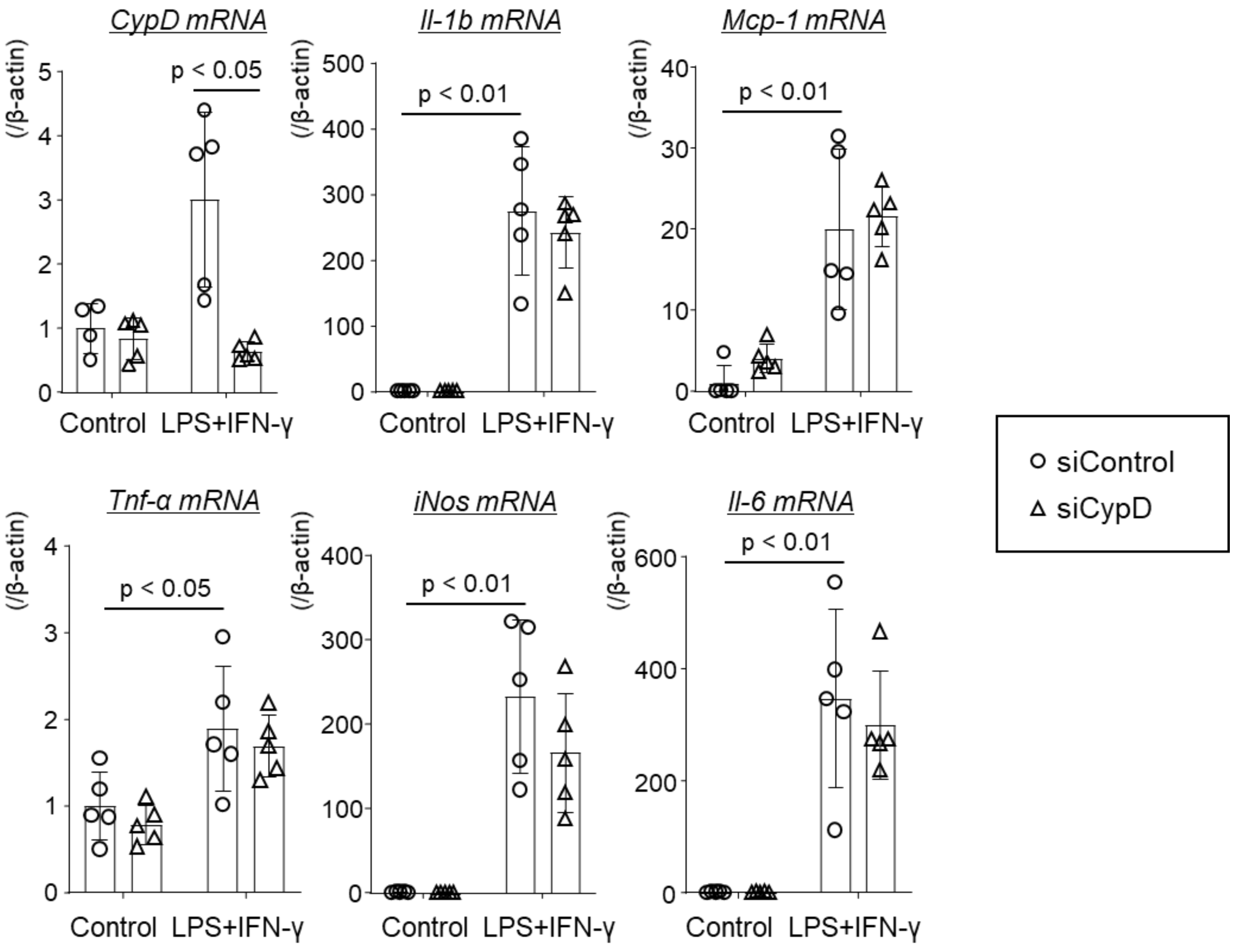

Supplemental Figure 1. siRNA-mediated knockdown of CypD in RAW264.7 cells. RAW264.7 cells were treated with 10 ng/mL and 10 ng/mL IFN-γ for 24 hours. siControl; control scramble siRNA, siCypD: CypD targeting siRNA. N=5.

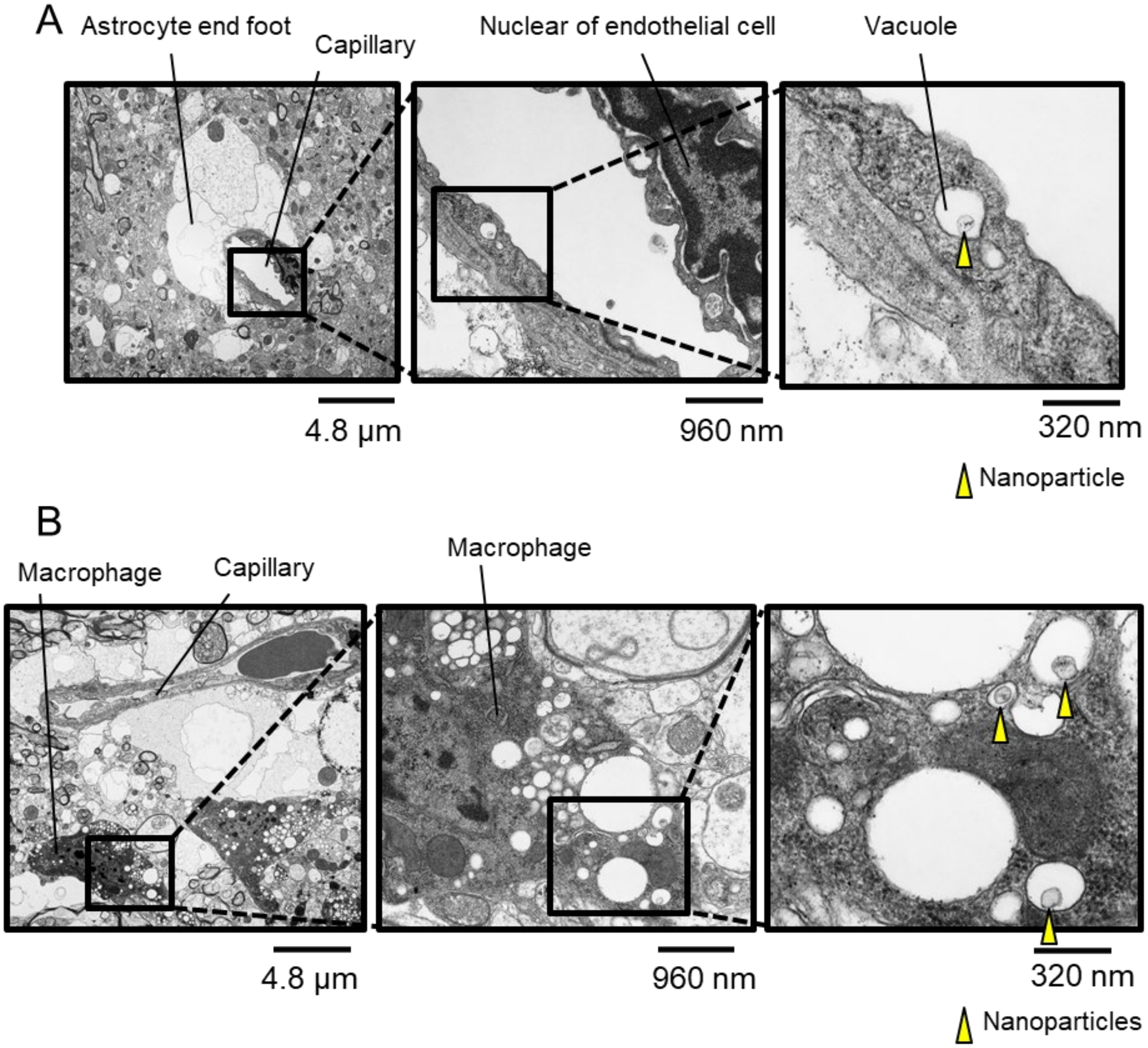

Supplemental Figure 2. Electron microscopic images of the brain tissue acquired 6 hours after IR injury. FITC-NPs (containing 4 mg/kg FITC) were intravenously injected at the time of reperfusion. PLGA-NPs were observed in vesicular structures of vascular endothelial cells (A) and macrophages (B). Arrowhead indicates PLGA-NP.

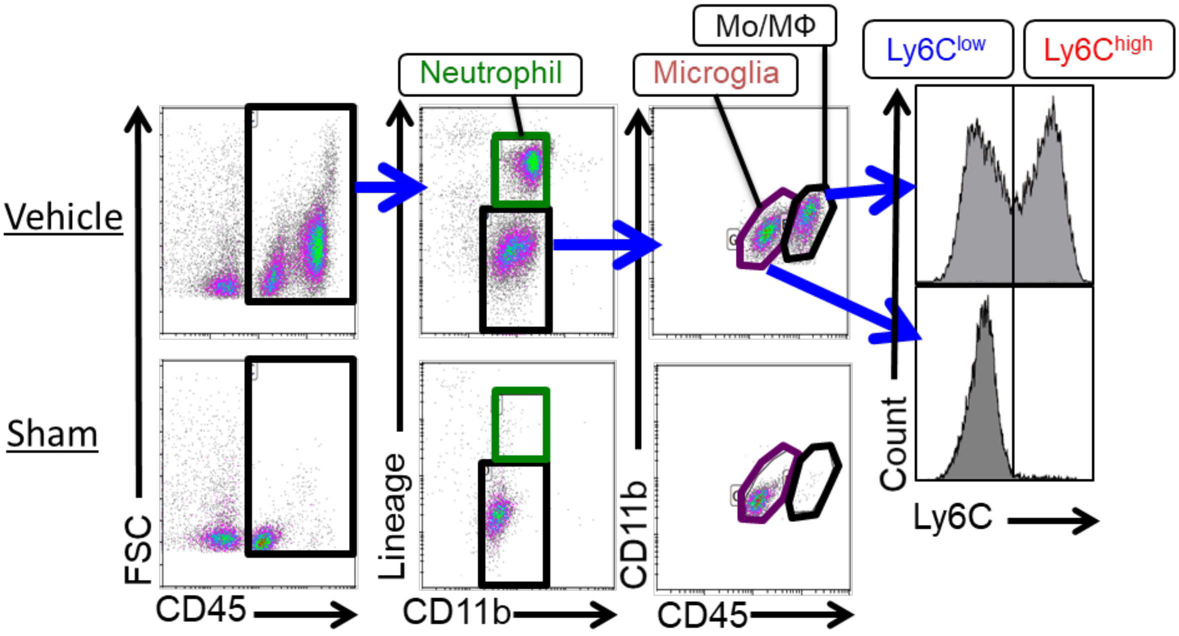

Supplemental Figure 3. Flow cytometric gating strategy for the neutrophil, microglia and monocyte/macrophage populations.
